# Cognitive behavioral therapy of socially phobic children focusing on cognition: a randomised wait-list control study

**DOI:** 10.1186/1753-2000-5-5

**Published:** 2011-02-28

**Authors:** Siebke Melfsen, Martina Kühnemund, Judith Schwieger, Andreas Warnke, Christina Stadler, Fritz Poustka, Ulrich Stangier

**Affiliations:** 1 Clinic and Polyclinic for Psychiatry, Psychosomatic and Psychotherapy for Children and Adolescents, University of Wuerzburg, Fuechsleinstr. 15, 97080 Wuerzburg, Germany; 2 Department of Child and Adolescent Psychiatry, University of Zurich, Switzerland; 3 University of Frankfurt, Department of Psychology, Germany; 4 Clinic and Polyclinic for Psychiatry and Psychotherapy for Children and Adolescents, University of Frankfurt, Germany

## Abstract

**Background:**

Although literature provides support for cognitive behavioral therapy (CBT) as an efficacious intervention for social phobia, more research is needed to improve treatments for children.

**Methods:**

Forty four Caucasian children (ages 8-14) meeting diagnostic criteria of social phobia according to the Diagnostic and Statistical Manual of Mental Disorders (4^th ^ed.; APA, 1994) were randomly allocated to either a newly developed CBT program focusing on cognition according to the model of Clark and Wells (n = 21) or a wait-list control group (n = 23). The primary outcome measure was clinical improvement. Secondary outcomes included improvements in anxiety coping, dysfunctional cognitions, interaction frequency and comorbid symptoms. Outcome measures included child report and clinican completed measures as well as a diagnostic interview.

**Results:**

Significant differences between treatment participants (4 dropouts) and controls (2 dropouts) were observed at post test on the German version of the Social Phobia and Anxiety Inventory for Children. Furthermore, in the treatment group, significantly more children were free of diagnosis than in wait-list group at post-test. Additional child completed and clinician completed measures support the results.

**Discussion:**

The study is a first step towards investigating whether CBT focusing on cognition is efficacious in treating children with social phobia. Future research will need to compare this treatment to an active treatment group. There remain the questions of whether the effect of the treatment is specific to the disorder and whether the underlying theoretical model is adequate.

**Conclusion:**

Preliminary support is provided for the efficacy of the cognitive behavioral treatment focusing on cognition in socially phobic children. Active comparators should be established with other evidence-based CBT programs for anxiety disorders, which differ significantly in their dosage and type of cognitive interventions from those of the manual under evaluation (e.g. Coping Cat).

## Background

Social phobia is one of the most common psychological disorders in children and adolescents [1-3]. The disorder is characterized by a fear of being perceived as inadequate in social or achievement situations, resulting in considerable problems. Furthermore, social phobia in childhood and adolescence is a risk factor for the development of other psychological disorders [4]. Although literature provides support for cognitive behavioral therapy (CBT) as an efficacious intervention for social phobia in children and adolescents [5-7], more research is needed to improve treatments for children. Most of the initial investigations included children with various anxiety disorders.

Kendall [8] developed the "Coping Cat program (Cat)" that contains education, modification of negative cognitions, exposure, social competence training, coping behavior and self-reinforcement. Different authors have used the program, making only slight changes [e.g. [9,10]]. Kendall [8] reports significantly less general anxiety and improved coping behaviour as a result of the program, even in a follow-up after 3.5 years [11].

"Cognitive-behavioral group therapy for social phobia in adolescents (CBGT-A)" [12], is a specific group program. The first phase conveys information about social phobia, and implements cognitive restructuring and social skill training. The second phase includes in vivo exposure and applied routines. Studies have demonstrated improvements at post test [13]. However, gains were not maintained at a 1-year follow-up [14].

The group program "Social effectiveness therapy for children" (SET-C) [15] puts its focus on exposure treatment, combined with social skills training and social interactions with non-anxious peers, but does so without cognitive interventions. Children and adolescents complete one introductory educational session with their parents, 1 group session, and 12 in-vivo exposure sessions over a 12 week period to help them improve their social skills. The SET-C group sessions provide instructions and practice, including activities where socially anxious participants interact with non-anxious peers. The individual in-vivo exposure component is designed to reduce anxiety in destressing social situations by making them more familiar. Concurrently, parents use positive reinforcement and shaping sequencing to effectively assist the progress of the SET-C program. Positive benefits have been achieved through use of this treatment protocol. Elements from the SET-C protocol were included in a school-based group behavioral treatment [15-19]. In one of the longest follow-up assessment studies on youth, Garcia-Lopez et al. [20] reported maintenance of treatment gains at the 5-year follow-up assessment. Masia et al. [18] built on this new approach in their investigation of a 14-session group treatment in a school-setting which focuses primarily on education, realistic thinking, social skills training, exposure, and unstructured social situations to allow for practicing skills. In a pilot study of six children, three of them no longer met criteria for social phobia [18]. Baer and Garland [21] used a modified version of the SET-C program. The treatment involved twelve sessions. The authors concluded that a briefer version of group CBT was as effective as the more extensive research protocols.

Several reseachers posit that cognition plays an important role in the maintenance of social phobia [22,23]. In an attempt to increase the overall response rate for cognitive-behavioral treatment, Clark and Wells [22] proposed a cognitive model of the maintenance of social phobia and used the model to develop a new cognitive therapy (CT) program for socially phobic adults. The four maintenance processes that are highlighted in the model are: (a) Increased self-focused attention; This means that in social situations, attention is shifted away from external social cues and instead is excessively self-focused. Connected with this is a linked decrease in observation of other people and their responses. (b) The use of misleading internal information (feelings and images) to make excessively negative inferences about how one appears to others. (c) Extensive use of overt and covert safety behaviors. Safety behaviors are strategies that are used to reduce anxiety or to hold off the social threat [24]. Safety behaviors, however, are problematic because they contribute to the maintenance of fear. Anticipatory as well as post-event thoughts (i.e. thoughts prior to and after the social situation) contribute to the persistence of social phobia. It was shown that the inclusion of interventions targeting safety behavior leads to an increased effectiveness of CBT [25]. (d) Problematic pre- and post-event processing [26]. The therapy program has proved to be superior compared to treatment with SSRIs or placebo, even after 12 months [26,27]. Higher effect sizes have been found compared to previous meta-analyses of cognitive-behavioral therapy in socially phobic adults. This result indicates a significant increase of effectiveness [26-28].

Very often, cognitive interventions are conceived as being inadequate for children due to their concrete thinking, time-limited perceptions and egocentric nature of thinking. It has, however, been suggested that children are quite capable of benefiting from cognitive interventions providing that educational and developmental features are considered. According to Ronen [29] children can benefit from cognitive interventions provided that two conditions are met: (1) The therapist should be able to adapt the treatment to the child's personal cognitive style. Such adaptations include, for example, translations of abstract terms to concrete ones, utilization of simple words, use of demonstrations, metaphors, and illustrations taken from the child's own day-to-day life. (2) The treatment goals and procedures should be suited to the child's individual pace, as related to age and cognitive level.

Hodson et al. [30] investigated the applicability of Clark and Wells' cognitive model to younger patients. High socially anxious children scored significantly higher than low socially children on all of the variables in Clark and Wells' model: negative social cognitions, self-focused attention, safety behaviours, and pre- and post-event processing. Findings suggest that Clark and Wells' model may be equally applicable to younger children with social phobia.

These findings have been confirmed by several studies [31-34]. Results from a range of studies show that anxious children interpret ambiguous situations more often as being hostile [35-37,31]. Muris et al. [38] showed a similar finding specifically with socially anxious children. Studies of attention control substantiate these findings: They confirm that the anxious child maintains a vigilant attention state for threatening cues [39-41]. Bell-Dolan and Emery [42] showed in a peer interaction task, that anxious children were as accurate as non-anxious children at identifying hostile intent in peer interactions, but they tended to misinterpret non-hostile situations as hostile. In a study by Johnson and Glass [43] socially anxious children, in social or evaluation situations, also tended to focus their attention primarily on themselves, for instance, on their own physical reactions, instead of on the business at hand. Very few studies have examined the memory capacity of anxious children. In a study by Daleiden [44] anxious children more often remembered negative information, so that a selective memory capacity was presumed to exist. In terms of anticipation of future events by socially anxious children, Spencer et al. [45] found with 7- 14 year olds that, in comparison to children in the control group, the socially anxious children underestimated the probability of future positive social events. Controlled studies of cognitive treatment programs for socially phobic children are rare.

Therapy with children differs from therapy with youth and adults. First, very few children come to therapy on their own volition. They are brought to treatment, usually by their parents or caregivers. Second, unlike adult therapy, which involves the rational modification of thoughts, cognitive behavioral therapy for children focusing on cognition is more concerned with teaching appropriate skills and applying certain techniques.

The following study deals with the evaluation of a new cognitive behavioral treatment program for socially phobic children focusing on cognition according to the model of Clark & Wells [22]. Although overlapping with other empirically validated CBT programs, CBT focusing on cognition has several distinctive features: (a) the development of Clark & Well's [22] model by using the child's own thoughts, images, attentional strategies, safety behaviors, and symptoms, (b) experiential exercises in which self-focused attention and safety behaviors are systematically manipulated in order to demonstrate their adverse effects, (c) systematic training in externally focused attention, (d) techniques for restructuring distorted self-imagery, including a specialized way of using video feedback and (f) the structuring of planned confrontation with feared social situations as a behavioral experiment in which children test pre-specified negative predictions while dropping their habitual safety behaviors and focusing externally. A habituation rationale was not used [26]. The aim of the present research was to examine the efficacy of this treatment program for socially phobic children with a focus on cognition. Our hypotheses include reduction of socially phobic symptoms and dysfunctional cognitions, improvements in anxiety coping, interaction frequency and comorbid symptoms.

## Methods

### Design

This was a single-center, parallel-group study with balanced randomization. Patients were randomly assigned to a cognitive behavioral treatment focusing on cognition or a wait-list control group. Children placed in the wait-list control group were offered the full treatment at the completion of the wait-list period. At three time-points in the study, treatment group participants completed questionnaires and diagnostic interviews: prior to beginning treatment, immediately following the final session and six months following termination of treatment. Wait-list participants completed measures at pre-test, after 4 months and after 10 months. Results of the follow-up data are in preparation. The ethics committee of the German Psychological Association (DGPs) had approved the project and written informed consent for the procedure was obtained from the children's parents. The program was delivered in and around Frankfurt am Main, Germany.

### Randomization

Patients were randomly assigned to intervention or control by using a web based computerised randomization plan generator http://www.randomization.com. The program randomizes each socially phobic child to a single treatment using the method of randomly permuted blocks. A research assistant not involved in the delivery of the treatment program placed participants on the randomization list in the next available slot.

### Participants

Forty four German socially phobic children and their respective mothers participated in the study. Children were recruited in and around Frankfurt am Main, Germany by means of advertisements and school contacts as well as through therapeutic institutions. The children were allocated to treatment on the basis of a computer generated random sequence. In the treatment group, there were 21 socially phobic children (Table 1). The control group consisted of 23 socially phobic children. The unequal size of both groups arose from the random allocation to the groups.

**Table 1:** Description of the children's sample

		treatment group (n = 21)	Wait-list group (n = 23)	
age	M (SD)	10.60 (1.64)	10.76 (1.90)	F(1,41) = .94, p = .33
	range	8 - 14	8 - 14	

gender	n (f/m)	8/13	13/10	Chi^2^(1, 0.95) = .91 p = .76

Caucasian	n	21	23	

Culture Fair Test				
	M (SD)	103.86 (13.41)	112.45 (12.23)	F(1,41) = .09 p = .09

comorbid diagnosis				
another anxiety disorders		n	10	7
affective disorder			1	0
enuresis			1	0
oppositional defiant disorder			0	1

drop-outs			4	2

### Measures

#### Intelligence

As a precondition for treatment, a measure of intelligence was administered in order to be able to exclude the possibility that differences in outcome measures could be attributed to differences in intelligence. The CFT-20 was administered to every child [46]. This intelligence test is the revised version of the "Culture Fair Test" and is adapted for the age range of 8, 5 to 18 years. Norms are constructed so that a person of average intelligence would reach an IQ value of 100. All four subtests showed high loads on the factor "General Fluid Ability". Correlations between CFT-20 and other intelligence tests have been found to be on average at a level of r = .64 with a range from r = .57 to r = .73 (see table 1).

#### Clinician-Completed Measures

All of the children took part in a structured interview for the diagnosis of mental disorders according to DSM-IV criteria. For this purpose, the German version of the Anxiety Disorders Interview Schedule (ADIS) for Children (German version: DIPS-K) [47,48] was administered. Previous research has demonstrated satisfactory interrater diagnostic reliability (r = .60) and test-retest reliability (kappa = .50) and the measure has shown sensitivity to treatment effects in studies of children and youth with anxiety disorders. Clinicians were trained by observing live and videotaped samples. They met an initial reliability criterion of 100% with the primary and comorbid diagnoses on five consecutive live child-parent interviews. Further, the child and parent interviews were videotaped. In order to get independent assessments, video recordings of all interviews at initial as well as outcome assessments were viewed by an expert who was blind to the treatment condition. The expert's ratings were final measures of the outcome.

##### Clinicians severity ratings

The DIPS-K contains rating scales (0-8-point) to assess the severity of disorder based on the clinicians' views of the degree to which the child's disorder(s) interfere(s) with overall functioning. Reliability for the clinician severity ratings has been found to be satisfactory (79% agreement was obtained).

##### Measure of overall functioning

Clinicians also completed the Children's Global Assessment Scale (K-GAS) [49], a clinician-rated scale that assesses overall functioning. The score can range between 1 and 100, with a lower score representing a more severe impairment. Interrater-reliability for the K-GAS was k = .85.

#### Child-Completed Measures

All of the scales presented in this study are validated scales.

##### Social Anxiety

The children were provided with the German version of the Social Phobia and Anxiety Inventory for Children (German version: SPAIK) [50,51]. The items refer to differences in frequency from 0 ("never, or hardly ever"), 1 ("sometimes") or 2 ("most of the time, or always" rated), with possible total scores ranging from 0 - 52. The SPAI-K appears to be a reliable (α = .92; r_tt _= .84) and valid measure (r = .6) of childhood social anxiety.

##### Anxiety coping

The German version of the "Coping Questionnaire - Child (German version: CQ-C)" [8] was developed to assess the child's self-perceived capability to deal with specific anxiety-provoking situations. Mother and child choose together 3 social situations in which the child experienced social fear. The child rated these on a five-point scale from "It is not difficult for me at all" (1) to "It is very difficult for me" (5). The test-retest reliability of the American version after two months in children with an anxiety disorder was given as r_tt _= .73 [8]. The German version has not been validated.

##### Dysfunctional cognitions

The German scale "Socially Anxious Cognitions Scale for Children (SAKK)" [52] was administered to assess socially anxious cognitions. The items are to be rated on a five-point scale with "never," "rarely," "sometimes", "mostly" or "always" as reponse options. It appears to be a reliable (α = .84-.91; r_tt _= .84) and valid measure (r = .64). Normative values for the SAKK are available for class levels 3-6.

##### Interaction frequency

A German behavior diary was implemented to assess social interactions. The frequency of telephone calls and activities with peers during a time period of 14 days was recorded in the diary. This measure builds on everyday behavior of children.

##### Comorbid symptoms

The Children's Depression Inventory (DIKJ) [53] is a German self-report measure of depressive symptoms. Severity of depressive symptoms is rated on a scale from 0 (not exists) to 3 (strong expression). Scores obtained on the DIKJ have been found to correlate significantly with clinicians' ratings of depression as well as with objective behavioral measures of depression. Internal consistency coefficients range from α = .82 through α = .91.

##### Treatment response

We used several different outcome measures. Our primary outcome measure was clinical improvement, assessed by a child-completed inventory (German version of the Social Phobia and Anxiety Inventory for Children). A second primary clinical outcome measure was the proportion of children who no longer met criteria for social phobia. Secondary outcomes included improvements in anxiety coping, dysfunctional cognitions, interaction frequency and comorbid symptoms.

### Procedure

#### Assessment and Diagnosis

**Figure 1:**
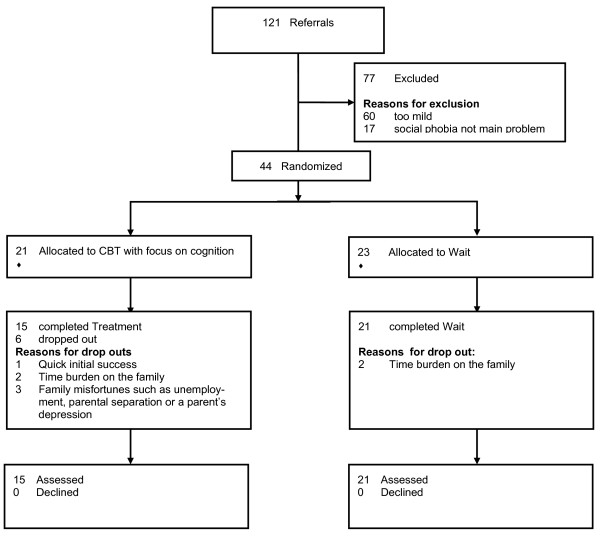
**Flowchart of patients' progress through phases of the trial**. DSM-IV = Diagnostic and Statistical Manual of Mental Disorders (4th ed.; American Psychiatric Association, 1994); CBT = cognitive behaviroal therapy, focus on cognition; WAIT = Wait-list control condition.

Two advanced doctoral level graduate students conducted all screening interviews as well as the implementation of the intervention. However, video recordings of all interviews at initial as well as outcome assessments were viewed by an expert who was blind to the treatment condition. The expert's ratings were final measures of the outcome. At the phone interview phase 121 children were assessed between 2004 and 2006 for possible inclusion in the trial. The DIPS-K was scheduled following initial phone contact with parents expressing interest in the study. The administration of the assessment measures was conducted in two separate sessions. This was done prior to beginning treatment as well as immediately following the final session (treatment group) and at 0 and 4 months after recruitment for the children on the wait-list. Because of limited capacity and the shorter attention span of children, assessment measures could not be performed in one session. During the first session, children and mothers were administered the DIPS-K and the questionnaires. Mother and child interviews were conducted separately and endorsement of the diagnostic criteria for social phobia by either mother or child was required for inclusion in the study. In the second session, children and parents completed the remaining questionnaires. 77 children were excluded (Figure 1 summarizes the reasons; additional file 1).

Children were offered inclusion if they met the following criteria: (a) the child met DSM-IV (American Psychiatric Association, 1994 [54]) criteria for social phobia, as defined by DIPS-K interview with mother and child; (b) the child had experienced social phobia for a duration of at least 6 months; (c) social phobia was considered to be the child's main current problem; (d) the child was 8 - 13 years old, and (e) the child and parents agreed not to start any additional treatment during the trial. Exclusion criteria for participation in the trial were psychotic symptoms, current suicidal or self-harming behavior or current involvement in other psychosocial or psychopharmacological treatment for phobia and anxiety problems. The exclusion criteria were assessed via interview (DIPS-K).

Children placed in a wait-list control group were offered the full treatment at the completion of the wait-list period. 17 of the 23 wait-list participants chose to attend these treatment sessions. The other six refused to participate. The reasons for refusal related to time burden of the parents and lack of motivation on the part of the socially phobic child.

#### Treatment

The treatment consisted of twenty 50-minute individual sessions and 4 parent sessions [55]. The individual sessions occured weekly. 20 treatment sessions represents a lengthy intervention. "Children" is far from a homogenous category, and treatments that ignore important developmental differences in child comptencies are likely to be too "generic" for optimal effectiveness [56]. Instead of group treatment, we used individual settings. A benefit of the one-on-one setting is a stronger adjustment to the individual characteristics of the patient. Furthermore, children with very high social anxiety participate least in group work or avoid attendance altogether. Studies point out that in an individual setting, comparable [57] or even better [58,59] results can be achieved than in a group setting. The present treatment manual (see Table 2) does not include social-skills training. Social deficits do not seem to play a central role in social phobia [60,32]. Instructions on situation-specific social skills were given to four children before behavioral experiments were carried out.

**Table 2:** Content of the sessions

Session No.	Content	Material
1-5	psycho-education (goals: relationship to the child, the child's motivation, the externalization of anxiety, normalization of fears, information on social anxiety, target setting, creating an anxiety hierachy, strategies for overcoming fears)	Therapeutic story as part of each session, hand puppets, puzzles, pictures, songs, stories, games, information sheets about social anxiety

6-8	cognitive restructuring: negative thoughts in advance of social situations and subsequent re-evaluations	Picture stories, stories, games and encouragement to discourage 'bad' thoughts

9-18	Preparation of behavioral experiments with gradually increasing difficulty, assessment of safety and avoidance behavior, discussion of potential obstacles, attention training, behavioral experiments in vivo	Various role-playing, some with video feedback, "Angstopoly" (board game with the implementation of social practice)

19	Summary and conclusion of the therapy, dealing with relapses	

20	Booster Session	

Parents	Parent sessions: Information on social anxiety in children, video-based assessment for the caregivers on how to deal with the child's fears, information about behavioral experiments and possibilities for supporting the child Closing session	

#### The treatment pursued the following objectives

1. Education about social phobia, behaviours like avoidance and safety behaviours.

2. Externalisation of attention and regulation of attention towards task-specific aspects.

3. Verification of anxious beliefs such as misleading internal information (feelings and images) if they give up safety behaviors.

4. Cognitive restructuring, differenciating anticipatory and post-event thoughts.

**The following interventions were used to implement the objectives **(for more details see additional file 2: Appendix A):

Therapy with children is generally based on an experimental here-and-now-approach. Children learn by doing. Action in therapy is enlivening. Children's motivation increases when they are having fun [61].

1: The therapist elicits information concerning the development of social phobia, situational determinants and temporal course. Several child-friendly techniques which make use of multiple sensory modalities are administered, e.g. drawing, songs, puppet play, games, storytelling, use of metaphors and craft work. These techniques add fun to therapy with children, increasing the reinforcing value of the sessions.

2: Attention training exercises enhance the shifting of socially phobic children's attention from themselves to the social situation in order to learn the externalisation of attention and the regulation of attention towards task-specific aspects to ease the intake of corrective information from the environment.

3: Behaviour experiments are implemented. Role plays with video feedback are used as preparation for the behavior experiments. Explicit reinforcement is a central part of our work with socially phobic children.

4: Furthermore, the child has to recognise unhelpful and anxiety-provoking self-statements and expectations in relation to social interactions.

All sessions were videotaped, and a sample of 25% of the sessions was selected for review in order to determine adherence to the treatment protocol. The treatment was carried out from 2004 to 2007.

### Statistical Analysis

#### Statistical Power

Results of studies exploring the effectiveness of cognitive treatment programs in socially phobic patients [27,28] available at the time of the study were used for power analyses. These studies demonstrated a high effect size for outcome measures (d = 1.2 - 2.4). The analyses indicated that for power = 90 with an alpha = 0.05, 20 participants per group would be required for child outcome measures. Given the expected high rate of drop-outs and loss for participants in the study, the number of participants recruited to the intervention and the wait-list groups was increased to 46, ensuring that the required sample size was achieved.

#### Statistical Analysis

All statistical analyses were conducted using SPSS 14.0. Intervention efficacy was assessed by comparing the outcomes of the wait-list control and the intervention condition at post-test. Missing outcome data were imputed. Analyses were intention-to-treat with the last available data point carried forward, if necessary. In order to identify any differences between the CBT treatment focusing on cognition and the wait-list, we compared scores for both groups using one-way analyses of variance (ANOVAs) for the primary outcome measure and for all secondary outcome measures. Potential confounds (e.g. socioeconomic status) and moderators (e.g. child gender) were explored.

The proportion of participants who no longer met criteria for the social phobia diagnosis at post-test in the two conditions was examined using χ^2 ^tests of independence.

Effect sizes are given as Hedges' G throughout the paper. Like Cohen's d, Hedges G is calculated by dividing the difference between treatment and wait list control group means at endpoint by the pooled standard deviation, but it uses a slightly different formula to calculate the latter, correcting for biases that can occur in smaller sample sizes [62]. To describe the magnitude of effect sizes, we have used criteria from Cohen [63]. Cohen [63] proposed a threefold classification of effect sizes: small (0.20 - 0.49), medium (0.50 - 0.79), and large (0.80 and above).

## Results

### Characteristics of Patients

The patients' mean age was 10.60 (SD = 1.64) in the treatment group and 10.76 (SD = 1.90) in the wait-list group, with an age range from 8 to 14 years. All patients had the generalized subtype of social phobia. In the treatment group there were 8 girls and 13 boys, in the wait-list group there were 13 girls and 10 boys. The main comorbid disorders were other current anxiety disorders (treatment group: n = 10; wait-list group: n = 7) (Table 1). Four patients in the treatment group and 2 patients in the wait-list group were classified as dropouts.

### Pre-treatment differences between groups

To determine the presence of pre-existing differences between participants in the wait-list and treatment group, a series of independent samples t-tests (for interval or ratio data), chi-square analyses (for nominal data) and ANOVAS were conducted (Table 3). The treatment and control groups were comparable with respect to age (F(1,41 = .94 p = .33), gender χ^2^(1, 0.95) = .91 p = .76) and intelligence (F(1,41) = .09 p = .09) assessed with the CFT-20. Participants in the treatment and control groups did not differ in terms of initial severity and psychopathology as assessed by the K-GAS (F(1,42) = .49 p = .58), SPAIK (F(1,42) = 3.71 p = .06), CQ-C (F(1,42) = .01 p = .94), DIKJ (F(1,42) = .68 p = .41), and behavior diary (F(1,32) = .50 p = .48) with all p > .05. However, the wait-list group showed a significantly higher SAKK-score for the subscale "negative self-evaluation" (F (1, 28) = 12.77, p < .001) and a lower SAKK-score for the subscale "positive self-evaluation" (F (1, 28) = 12.99, p < .001). There were no differences between dropouts and participants in demographic variables.

**Table 3:** Effects of CBT focusing on cognition for primary and secondary outcome measures across time

	Treatment group (n = 21)	Wait list (n = 23)	
	M (SD)	M (SD)	Group effect
CHILD-COMPLETED PRIMARY OUTCOME MEASURES			

Social Phobia and Anxiety Inventory for Children, German version (SPAIK)			
Pre-treatment	24.47 (7.23)	20.60 (6.09)	F(1,42) = 3.71 ns
Post-treatment	12.30 (9.13)	18.41 (8.53)	F(1,42) = 5.26 *

CLINICIAN-COMPLETED PRIMARY OUTCOME MEASURES			

Severity (DIPS-K)			
Pre-treatment	5.33 (1.24)	5.17 (0.58)	F(1,42) = .31 ns
Post-treatment	3.43 (2.44)	4.96 (0.42)	F(1,42) = 6.33*

CHILD-COMPLETED SECONDARY OUTCOME MEASURES			

Coping Questionnaire - Child (CQ-C)			
Pre-treatment	3.11 (0.62)	3.10 (0.57)	F(1,42) = .01 ns
Post-treatment	1.77 (1.19)	2.27 (0.89)	F(1,42) = 2.57 ns

Socially Anxious Cognitions Scale for Children (SAKK)			
Positive Self-evaluation			
Pre-treatment	19.83 (7.67)	13.23 (6.64)	F(1,37) = 8.21**
Post-treatment	24.52 (8.14)	14.98 (6.11)	F(1,35) = 16.56***
			
Negative Self-evaluation			
Pre-treatment	8.85 (6.14)	13.68 (6.29)	F(1,37) = 5.90*
Post-treatment	7.78 (6.26)	12.15 (7.23)	F(1,36) = 3.92*
			
Coping ideas			
Pre-treatment	14.25 (6.33)	11.89 (7.73)	F(1,37) = 1.09 ns
Post-treatment	17.68 (7.02)	11.94 (6.16)	F(1,38) = 7.60**

Behavior Diary			
Pre-treatment	18.72 (7.63)	20.50 (6.88)	F(1,32) = .50 ns
Post-treatment	19.21 (7.55)	19.84 (6.49)	F(1,36) = .076 ns

Children's Depression Inventory (DIKJ)			
Pre-treatment	11.52 (6.87)	9.91 (6.06)	F(1,42) = .68 ns
Post-treatment	9.71 (9.06)	11.22 (6.80)	F(1,42) = .39 ns

CLINICIAN-COMPLETED SECONDARY OUTCOME MEASURES			

Overall functioning			
Pre-treatment	52.14 (7.84)	53.70 (6.94)	F(1,42) = .49 ns
Post-treatment	61.19 (14.31)	55.43 (5.62	F(1,42) = 3.19 p = .08

Note: * p < .05; ** p < .01: *** p < .001 ns not significant; scores for both groups were compared with one-way analyses of variance (ANOVAs) for the primary outcome measure and for all secondary outcome measures

#### Effects of Treatment on Social Phobia

##### Primary outcome results

##### Child-completed measures (Table 3)

Analysis of the child-completed measures indicated that CBT focusing on cognition was associated with significant pre-treatment-to-post-treatment improvement. The Social Phobia and Anxiety Inventory for Children (SPAIK) showed a significant decrease in social phobia symptoms (F(1,42) = 5.26 p ≤ .05). No harm occured.

##### Clinician-Completed Measures (Table 3)

At the post-treatment assessment, social phobia was assessed in all children on the wait-list group. In the treatment group, seven of the children no longer showed social phobia, 10 of the children significantly improved, 4 other children had been dropouts. This difference was significant (χ^2 ^(1, 0.95) = 12.0714, p ≤ .001).

Hedges G [62] was used to calculate effect sizes comparing the treatment with the wait-list condition. The measures of social phobia showed medium to large effect sizes (clinician social phobia severity ratings, DIPS-K: G = 0.89, SPAIK: G = 0.94).

##### Secondary outcome results

##### Child-completed measures (Table 3)

Significant improvements were observed in the inventory assessing dysfunctional cognitions (SAKK): The children from the CBT treatment group showed a significant increase in positive self-evaluation (F(1,35) = 16.56 p ≤ .001) and coping ideas (F(1,38) = 7.60 p ≤ .01) and a significant decrease in negative self-evaluation (F(1,36) = 3.92 p ≤ .05). The inventory assessing dysfunctional cognition (SAKK) showed large effect sizes: Positive Self-evaluation: G = 1.34, Negative Self-evaluation: G = 1.41; coping ideas: G = 0.86).

No significant changes were found in the behavior diary assessing interaction frequency (F(1,36) = .08 p = .78), in the Coping Questionnaire (CQ-C) (F(1,42) = 2.57 p = .12) and in the Depression Inventory for Children (DIKJ) (F(1,42) = .39 p = .54).

##### Clinician-Completed Measures (Table 3)

There was no significant difference, but a tendency towards improvement (F(1,42) = 3.19, p = .08) in overall functioning between pre-treatment and post-treatment, as assessed by the K-GAS.

## Discussion

The objective of this therapy efficacy study was to determine whether socially phobic children in the treatment group differed from socially phobic children in the wait-list group at the end of a newly developed cognitive behavioral therapy program focusing on cognition. The innovation of the newly developed treatment consisted in the following: (a) using the child's own thoughts, images, attentional strategies, safety behaviors, and symptoms, (b) systematic manipulation of self-focused attention and safety behaviors, (c) systematic training in externally focused attention, (d) techniques for restructuring distorted self-imagery and (f) behavioral experiments in which a habituation rational was not used.

Three important conclusions can be drawn from the study:

1) The study provides preliminary evidence that the outcome of CBT focusing on cognition is better than the natural course of the condition. At post-assessment, children who received CBT treatment focusing on cognition compared to children in the wait-list group showed a significantly greater decrease of social phobia symptoms on the Social Phobia and Anxiety Inventory for Children (SPAIK). Significant improvement could also be seen on the severity ratings (DIPS-K). All children from the CBT treatment group showed a lower severity of social phobia compared to the waitlist group after the treatment. In addition, 30% of the children in the treatment group were free of diagnosis after treatment, whereas in the waitlist group all of the participants held their diagnosis. This suggests that the CBT treatment focusing on cognition was able to produce clinical improvement in our sample of socially phobic children. However, recent review articles have concluded that CBT packages result in around 56% of children being free of either the principal or any anxiety disorder after treatment [64]. Therefore, reduction of anxiety diagnoses at posttreatment of our study was not within the range of those reported in CBT trials of children with different anxiety disorders.

2) Participation in our therapy decreased anxiety symptoms of social phobia and related symptoms such as negative feelings of self-worth. The results showed that the prevalence of comorbid symptoms like self-reported depression was not reduced as much as core symptoms by the treatment. However, we did not test whether symptoms of other anxiety disorders were also reduced. Further studies should examine whether the effect of the treatment was specific to the disorder of social phobia.

3) Decreased dysfunctional cognition as assessed by the SAKK suggests that the young children benefiting from our study were developmentally prepared to participate in a cognitive behavioral treatment focusing on cognition. Results from the Socially Anxious Cognitions Scale for Children (SAKK) with its Subscale of Negative Evaluation, Subscale of Positive Evaluation and Subscale of Coping Ideas, corroborate the overall results. Large effect sizes could be seen in this inventory (SAKK): g = 1.34 for Positive Self-Evaluation, g = 1.41 for Negative Self-evaluation and g = 0.89 for Coping Ideas.

Despite improvement in positive symptoms there was no improvement in K-GAS and behaviour diary ratings. There seems to be an inconsistency between positive symptom improvement but lack of functional improvement. However, changes of interaction may follow positive symptom improvement. The follow-up study will show whether such improvements may be observed.

### Limitations

The study represents a first step to clarify whether CBT with a focus on cognition is an effective therapeutic approach in the treatment of socially phobic children. Further studies are necessary, however, to investigate whether the results can be replicated and whether the underlying theoretical model is adequate for socially phobic children. The significant results in the inventory assessing dysfunctional cognition show preliminary evidence, but have to be supported in further studies. Further studies are also needed to examine whether CBT focusing on cognition is superior or comparable to a general CBT approach and to examine which therapeutic approach is better suited to which patients.

One of the study's major limitations is that two advanced doctoral level graduate students conducted all screening interviews as well as the administration of the intervention. As the children should not be unduly burdened, assessment and intervention were thus carried out by the same person. Consequently, there is no independent assessment. Therefore, on the one hand, there is the risk that the children responded in ways to please the familiar interviewer. On the other hand, however, unfamiliar interviewers are likely to cause social anxiety. It follows that socially phobic children very often would indicate less social anxiety by avoiding to talk to interviewers who are unfamiliar to them. However, video recordings of all interviews were reviewed by an expert who was blind to the treatment condition.

Another major limitation concerns treatment design. Similar to many first trials of new CBT protocols for anxiety, we conducted this initial trial using a wait-list control condition. This approach provides preliminary evidence that the outcome of the proposed intervention is better than the natural course of the condition. It should be further evaluated against other interventions in subsequent trials.

Furthermore, the trial has not been registered.

Six patients dropped out of our study, four of whom participated in the treatment group. However, compared to drop-out rates in other studies, the rate of drop-out in the present treatment program is not noticeably high: According to Lincoln [65] and Turner et al. [66], only approximately 40% to 50% of the socially phobic adult patients seeking treatment actually completed and benefited from it in the end. There are further problems in the treatment of children, as not only the child must be motivated to participate in the treatment. According to the parents, therapies were discontinued for various reasons: quick initial successes, which seemed sufficiently high, time burden on the family, family misfortunes such as unemployment, parental separation or a parent's depression led to the premature termination of their child's therapy. Thus, it was not always the children who were most impaired who dropped out and did not receive treatment. It could be also possible that a 20-session intervention may be too intensive for some participants.

Considering a waiting period of many months, a selective dropout could have affected the configuration of the control group: Rejection could have been perceived before the beginning of the study as well as during the waiting period. However, the dropout rate does not confirm this argument, as there were only 2 dropouts in the control group compared to 4 drop-outs in the treatment group. Presumably, this relates to the very difficult state of care facilities that provide psychotherapy for children and adolescents.

## Conclusions

Preliminary support is provided for the efficacy of a newly developed CBT treatment with a focus on cognition. Results from the clinician-completed and child self-report measures after the treatment are satisfactory. Future research will need to compare the treatment to another active treatment. Wait-list control has been argued to not be a true comparative control group as it may not produce a placebo effect. A study with an active treatment group is needed in order to determine whether the additional cognitive elements were superior or comparable to conventional CBT.

## Competing interests

The authors declare that they have no competing interests.

## Authors' contributions

SM, MK and JS carried out studies and drafted the manuscript. AW and US have made substantial contributions to conception and design. CS and FP have made substantial contribution to acquisition of data. All authors read and approved the final manuscript.

## Supplementary Material

Additional file 1 **CONSORTchecklist**. information on the manuscript according to the CONSORT checklist. Click here for file

Additional file 2 **Appendix A: Cognitive behavioral therapy of socially phobic children focusing on cognition**. Information on the treatment course. Click here for file
