# Supplementary material for: Cognitive behavioral therapy of socially phobic children focusing on cognition: a randomised wait-list control study
Source: Child Adolesc Psychiatry Ment Health. 2011 Feb 28;5:5. doi: 10.1186/1753-2000-5-5 (PMC3058082; doi:10.1186/1753-2000-5-5)
Supplement: Additional file 2 — Appendix A: Cognitive behavioral therapy of socially phobic children focusing on cognition. Information on the treatment course. [file 1753-2000-5-5-S2.DOC]

**Appendix A: Cognitive behavioral therapy of socially phobic children focusing on cognition**

### Session 1: Psychoeducation – information about social anxiety

|  | Contents | Material |
| --- | --- | --- |
| Beginning | Talk about likes, hobbies etc. | Puppets |
| **Motivation** | Introduction to the topic of anxiety management | Fear song |
| **Main part**  **Phase 1**  **Phase 2**  **Phase 3** | Description of a first therapy session from the perspective of another child – part 1  Introduction of the fear monsters  Awareness of different fears | Story as a therapeutic framework – part 1  Pictures  Crossword |
| **Phase 4** | Description of a first therapy session from the perspective of another child – part 2 | Story as a therapeutic framework – part 2 |
| **Completion** | Summary | Puppets |

### Session 2: Psychoeducation – information about social anxiety

|  | Content | Material |
| --- | --- | --- |
| Beginning | Review of the last week | puppets  fear song |
| **Motivation** | Introduction to the topic of fear“ | feel bag with anxiety monster |
| **Main part**  **Phase 1**  **Phase 2**  **Phase 3**  **Phase 4**  **Phase 5**  **Phase 6** | description of social anxiety from the perspective of another child  body reactions to fear  behaviour  thoughts  Repeat: fear reaction  Description of social anxiety from the perspective of another child | story as a therapeutic framework – part 1  list of body reactions  body schema  social anxiety fact sheet  Story as a therapeutic framework – part 2 |
| **Completion** | Summary | Puppets |

## Session 3: Therapy goal setting

|  | Contents | Material |
| --- | --- | --- |
| Beginning | Review of the last week | Puppets  Fear song |
| **Motivation** | Miracle question |  |
| **Main part**  **Phase 1**  **Phase 2**  **Phase 3** | Development of treatment motivation in another child – part 1  Develop treatment goals  Therapeutic communication, how life will change if the child lack social anxiety | Story as a therapeutic framework – part 1  Painting  Create horoscope |
| **Phase 4** | Development of treatment motivation in another child – part 2 | Story as a therapeutic framework – part 2 |
| **Completion** | Summary | puppets |

## Session 4: Develop the therapeutic rationale

|  | Contents | Material |
| --- | --- | --- |
| Beginning | Review of the last week | Puppets  Fear song |
| **Motivation** | Concept of Fear circuit | Frog game |
| **Main part**  **Phase 1**  **Phase 2**  **Phase 3** | Development of a fear circuit – part 1  Develop treatment goals  Repetition of the fear circuit: Child expains the fear circuit to another child | Story as a therapeutic framework – part 1  Picture of fear circuit  puppets |
| **Phase 4**  **Phase 5** | Deriving strategies to conquer anxiety from the circuit of fear  Development of a fear circuit – part 2 | Story as a therapeutic framework – part 2 |
| **Completion** | Summary | Puppets |

## Session 5: Strategies against anxiety

|  | Contents | Material |
| --- | --- | --- |
| Beginning | Review of the last week | Puppets  Fear song |
| **Motivation** | Repetition of the fear circuit | Puzzle of the fear circuit |
| **Main part**  **Phase 1**  **Phase 2** | First exposition – part 1  Strategies for dealing with fear:  a. avoidance  b. safety behaviors | Story as a therapeutic framework – part 1 |
| **Phase 4** | First exposition – part 2 | Story as a therapeutic framework – part 2 |
| **Completion** | Summary | Puppets |

## Session 6: Cognitive restructuring - 1

|  | Contents | Material |
| --- | --- | --- |
| Beginning | Review of the last week | Puppets |
| **Main part**  **Phase 1**  **Phase 2**  **Phase 3** | Assessment of thoughts  Categorization of thoughts  Effects of encouraging and frightening thoughts | Story of a thought collector  Cards with encouraging and frightening thoughts  Role playing  Comic about test anxiety |
| **Phase 4** | Collect encouraging and frightening thoughts |  |
| **Completion** | Summary | puppets |

## Session 7: Cognitive restructuring - 2

|  | Contents | Material |
| --- | --- | --- |
| Beginning | Review of the last week | Puppets |
| **Main part**  **Phase 1**  **Phase 2**  **Phase 3** | Discussion of homework  Change frightening into encouraging thoughts  Consequences of encouraging and frightening thoughts | Comic „Read aloud“  Cards with encouraging and frightening thoughts  „Story with a hammer“ by Watzlawik |
|  |  |  |
| **Completion** | Summary | Puppets  Comic „Birthday party“ |

## Session 8: Cognitive restructuring - 3

|  | Contents | Material |
| --- | --- | --- |
| Beginning | Review of the last week | Puppets |
| **Main part**  **Phase 1**  **Phase 2**  **Phase 3** | Discussion of homework  Post-mortem thoughts (i.e. thoughts after the social situation)  Collecting post-mortem thoughts | Comic „Birthday party“  Roleplay  Comic „The lecture“ |
|  |  |  |
| **Completion** | Summary | Puppets |

## Session 9: Preparation of behavioral experiments - 1

|  | Contents | Material |
| --- | --- | --- |
| Beginning | Review of the last week | Puppets |
| **Main part**  **Phase 1**  **Phase 2**  **Phase 3**  **Phase 4**  **Phase 5**  **Phase 6**  **Phase 7** | Overcoming fears  Selection of an anxiety situation to be processed  Avoidance and safety behavior  Collecting post-mortem thoughts  Roleplays with video feedback  Overcoming fears  Repetition | Story as a therapeutic framework – part 1  Story as a therapeutic framework – part 2  Game „Angstopoly“ |
| **Completion** | Summary | Puppets |

## Session 10: Attention training - 1

|  | Contents | Material |
| --- | --- | --- |
| Beginning | Introduction: Importance of attention training | Puppets |
| **Main part**  **Phase 1**  **Phase 2**  **Phase 3** | Acoustic exercises  Speaking exercises  Perception exercises |  |
| **Completion** | Summary | Puppets |

## Session 11: Behavioral experiment in vivo - 1

|  | Contents | Material |
| --- | --- | --- |
| Beginning | Review of the last week | Puppets |
| **Main part** | Behavioral experiment |  |
| **Completion** | Summary | Puppets |

## Session 12: Preparation of behavioral experiments - 2

|  | Contents | Material |
| --- | --- | --- |
| Beginning | Review of the last week | Puppets |
| **Main part**  **Phase 1**  **Phase 2**  **Phase 3**  **Phase 4**  **Phase 5**  **Phase 6**  **Phase 7** | Overcoming fears  Selection of an anxiety situation to be processed  Avoidance and safety behavior  Collecting post-mortem thoughts  Roleplays with video feedback  Overcoming fears  Repetition | Story as a therapeutic framework – part 1  Story as a therapeutic framework – part 2  Game „Angstopoly“ |
| **Completion** | Summary | Puppets |

## Session 13: Attention training - 2

|  | Contents | Material |
| --- | --- | --- |
| Beginning | Introduction: Importance of attention training | Puppets |
| **Main part**  **Phase 1**  **Phase 2**  **Phase 3** | Acoustic exercises  Speaking exercises  Perception exercises |  |
| **Completion** | Summary | Puppets |

## Session 14: Behavioral experiment in vivo - 2

|  | Contents | Material |
| --- | --- | --- |
| Beginning | Review of the last week | Puppets |
| **Main part** | Behavioral experiment |  |
| **Completion** | Summary | Puppets |

## Session 15: Preparation of behavioral experiments - 3

|  | Contents | Material |
| --- | --- | --- |
| Beginning | Review of the last week | Puppets |
| **Main part**  **Phase 1**  **Phase 2**  **Phase 3**  **Phase 4**  **Phase 5**  **Phase 6**  **Phase 7** | Overcoming fears  Selection of an anxiety situation to be processed  Avoidance and safety behavior  Collecting post-mortem thoughts  Roleplays with video feedback  Overcoming fears  Repetition | Story as a therapeutic framework – part 1  Story as a therapeutic framework – part 2  Game „Angstopoly“ |
| **Completion** | Summary | Puppets |

## Session 16: Attention training - 3

|  | Contents | Material |
| --- | --- | --- |
| Beginning | Introduction: Importance of attention training | Puppets |
| **Main part**  **Phase 1**  **Phase 2**  **Phase 3** | Acoustic exercises  Speaking exercises  Perception exercises |  |
| **Completion** | Summary | Puppets |

## Session 17: Behavioral experiment in vivo - 3

|  | Contents | Material |
| --- | --- | --- |
| Beginning | Review of the last week | Puppets |
| **Main part** | Behavioral experiment |  |
| **Completion** | Summary | Puppets |

## Session 18: Behavioral experiment in vivo - 4

|  | Contents | Material |
| --- | --- | --- |
| Beginning | Review of the last week | Puppets |
| **Main part** | Behavioral experiment |  |
| **Completion** | Summary | Puppets |

## Session 19: Emergency case, Memory case

|  | Contents | Material |
| --- | --- | --- |
| Beginning | Introduction: Relapses | Puppets |
| **Main part**  **Phase 1**  **Phase 2** | Social situations causing fears  Strategies to overcome the fears |  |
| **Completion** | Summary | Courage Plaque |

## Session 20: Booster session

|  | Contents | Material |
| --- | --- | --- |
| Beginning | Review of the last months | Puppets |
| **Main part**  **Phase 1**  **Phase 2** | Discussion of difficult social situations  Repetition of social exercises |  |
| **Completion** | Summary | certificate |
